# Editorial: Bacteriophages Isolation From the Environment and Their Antimicrobial Therapeutic Potential

**DOI:** 10.3389/fmicb.2021.649334

**Published:** 2021-02-23

**Authors:** Krishna Mohan Poluri, Sutthirat Sitthisak, Krishna Khairnar, Robert Czajkowski

**Affiliations:** ^1^Department of Biotechnology and Centre for Nanotechnology, Indian Institute of Technology Roorkee, Roorkee, India; ^2^Department of Microbiology and Parasitology, Faculty of Medical Science, Naresuan University, Phitsanulok, Thailand; ^3^Environmental Virology Cell (EVC), Council of Scientific & Industrial Research- National Environmental Engineering Research Institute (NEERI), Nagpur, India; ^4^Division of Biologically Active Compounds, Intercollegiate Faculty of Biotechnology, University of Gdansk and Medical University of Gdansk, University of Gdansk, Gdańsk, Poland

**Keywords:** bacterial viruses, phage (bacteriophage), isolation, biological control, medicine, veterinary, agriculture

First bacterial viruses (named bacteriophages and/or phages—“eaters of bacteria”) were discovered only as late as at the beginning of the twentieth century (Hadley, 1928; Abedon, 2014). Just after, since the early thirties of twentieth century, bacteriophages were widely used in the control and prevention of bacterial infections in humans (Van der Vlught and Verbeek, 2008). However, the discovery at the same time of the first natural antibiotics (i.e., penicillin) prevented the further development of therapies based on the use of phages in most Western European countries as well as in the rest of the world. Only recently, due to the worldwide appearance and spread of the multi-drug resistant bacterial infections in humans, bacteriophages have been rediscovered as potential antibacterial therapies and have started to be widely used again.

Despite the recent recognition of bacteriophages as potential “golden bullet” for treating infections caused by multi-drug resistant bacterial pathogens, only very little work has targeted this subject in detail. To boost this important research field, the Research Topic “*Bacteriophages isolation from the environment and their antimicrobial therapeutic potential*” was developed with the aim of underlining, among others, the potential new therapeutic roles of bacterial viruses in treatment of human, animal and crop plant infections.

Public health safety and economic concerns being the thematic megatrends define the societal development index. Bacteriophages can occur naturally and feed upon certain specific bacteria for their nutritional needs. Hence, they act as a natural antibacterial agent for particular organism. In the current thematic issue, several reports that include isolation of the bacteriophages from the natural niches are included. A diverse hotspot for the bacterial existence and their intuitive host (phages) is sewage sites. Yang M. et al. could isolate two lytic bacteriophages from the sewage collected in China. The phages were found to have *Vibrio parahaemolyticus* as their particular host, which implicatively destine these phages to be a potential control agent for multi-drug resistant *V. parahaemolyticus*. Morphological examination using electron microscopy enabled the authors to classify the virions in *Siphoviridae* family on the basis of typical features like icosahedral head and long non-contractile tail. Genetic annotations were used to tag major structural and functional genes involved in phage structure, packaging, host lysis, DNA metabolism etc. Similar to sewages, the outlets from the hospitals also attract fair amount of consideration with respect to phage isolation. Peng et al. isolated a phage named TCUEAP1 that has *Elizabethkingia anopheles* as its host. The genome sequence analysis was performed to ascertain the exclusivity of phage. Additionally, in mice experiments, this phage showed high rescue rate after lethal bacteremia. Pacifico et al. studied and established the existence of coliphages in the blood, urine and tracheal aspirates of hospitalized patients. Upon classification, clinical isolates were found to belong to *Tunavirinae* sub family and to *Peduovirus* and *Tequintavirus* genera. These newly isolated phages were virulent with exception to some members belonging to *Peduovirus* that seem to reside as prophages. Imam et al. isolated and characterized so-called jumbo bacteriophage MIJ3 infecting *Pseudomonas aeruginosa*. The genome of the isolated virus contains genes coding for proteins of bacterial origin, not frequently present in bacteriophages *viz*. FtsH (ATP-dependent zinc metalloprotease)-like protein, and TilS–a putative lysidine synthase. The presence of bacterial genes in phage genomes may shed light on the bacteriophage-bacteria gene exchanges, spread of the bacterial genes in environment and size of the genetic pool that can be available for bacteria in nature.

Altered environmental conditions such as global warming have created substantial impact on environment. Due to significant change in the seasonal variation patterns, the opportunistic environmental bacteria have become more pathogenic. Kabwe et al. reported the isolation and characterization of lytic phage against *Aeromonas hydrophila*. This bacteria can survive in moist temperate environment, form biofilms, survive traditional disinfection methods and even disarm antibiotics; making it responsible for mortality rate as high as 46%. Another classical niche for the bacteria and the associated phages is the soil and forms the considerable ground for metagenomics study. Peters et al. isolated and characterized a phage from soil and found it specific to *Stenotrophomonas maltophilia*. This phage was named as DLP3 (second member in *Delepquintavirus* genus) and found to infect 22 out of 29 known strains of *S. maltophilia*. The authors also performed genome analysis thereby identifying the coding sequences, resistance modules and other important genomic aspects. The phage also exhibited lytic activity *in vivo*. Apart from the soil, water bodies also serve as an important ecosystem that show close knit association and companionship amongst microbes. Bio-accumulation of the nutrients in the water bodies lead to profuse growth of organism of lower strata. Cyanobacteria, lie at the base of the food chain operating in water and often present lethal threats to the aquatic life-forms as they lower the levels of dissolved oxygen. Yang F. et al. isolated a long-tailed cyanophage that can dwell on cyanobacteria from Lake Chaohu. The authors described the morphological features of this isolated phage Mic1 specific to *Microcystis aeruginosa* and performed genome and phylogenetic analyses. Common phylogenetic ancestry of DNA polymerase gene from Mic1 and mitochondria strikes a note common to ancestral relationships between cyanophages and their hosts.

Acquired antibiotic resistance due to horizontal gene transfer, during nosocomial infections, antibiotic misuse and frequent treatment prolapses, is a major concern in present scenario. Bacteriophages, viruses that can feed upon bacteria, are being tested for this purpose and yielding a better performance stats, worldwide. Several bacteriophages have been discovered are under testing to understand and cure the bacterial nuisances. Rising instances of antibiotic resistant *Acinetobacter baumannii* strains have created a worldwide alarming condition. Wang C. et al. once again characterized an already isolated phage (named vB_AbaM_IME285). Recombinant enzyme (Dp49), a product of depolymerase gene (ORF49) from this phage, showed significant antibiotic activity *in vitro*. Increased survival rate of the mice pre-infected with *A. baumannii* projects this phage to be suitable alternative to control multidrug-resistant *A. baumannii*. Likewise, Rouse et al. used a mouse model to assess the effectiveness of phage treatment against *A. baumannii* infections. In this proof-of-concept approach the authors demonstrated that the lytic bacterial viruses can be used with success as a potent and safe antibacterial agent against the pathogen and that exposure of animals to phages prior to infections with *A. baumannii* does not result in decrease of the observed antibacterial effects.

Another major clinical problem that can employ bacteriophage as an alternative antibacterial remedy is carbapenem-resistant *Klebsiella pneumoniae* (CRKP). By their formidable efforts, Li et al. could isolate 54 phages, corresponding to 54 clinical CRKP hosts. Interestingly, KPC-2 producing ST11 strain was the most abundant strain (88.9%) and a phage P545 from *Myoviridaes* was highly stable and inhibited the biofilm formation. Worldwide, *Yersinia enterolitica* is considered a troublesome food borne pathogen. Xue et al. isolated and tested Yersinia phage X1, which has broad host range covering almost half of the known strains. Exceptional ability of this phage to cross stomach and reach intestine with retained infectious ability, along with high stability and tolerance with respect to pH, temperature and enzymatic degradation; makes this phage a suitable candidate for oral administration. Intestinal histopathologic observations exhibited phenomenal improvement upon treatment with phage X1, and reduced the level of proinflammatory cytokines. Similarly, in another study, Wang S. et al. tested the phages to control ampicillin-resistant *Escherichia coli*. The authors demonstrated that overexpression of AmpC β-lactamase—an enzyme responsible for bacterial resistance to ampicillin, may promote infection of *E. coli* strains with phages that use an OmpA as a receptor for adsorption. Furthermore, this study demonstrated an interplay between phages and antibiotics in killing bacterial pathogens and may give important insights to the global effectiveness of therapies involving antimicrobials and bacterial viruses. An interesting example describing the study of phages infecting Gram-positive bacterial human pathogens is the work of Lee et al. who characterized a bacteriophage vB_EfaS_HEf13 expressing broad lytic activity against clinically-relevant antibiotic-resistant isolates of *Enterococcus faecalis*. The authors speculated that the mentioned bacteriophage may be potent as a dental therapeutic agent to treat periodontitis related to infections with *E. faecalis*. Furthermore, bacteriophages can be also used to prevent/reduce biofilm formation during catheterization of hospitalized patients. This approach has been exploited by Alves et al. who isolated lytic bacteriophages against *Proteus mirabilis—*a common causative agent of biofilm formation in catheters. The authors characterized in detail five phages able to infect *P. mirabilis*, including these that shown to reduce formation of biofilm under experimental conditions. Such an approach may be of help in developing new therapies for hospitalized individuals who require (permanent) catheterization.

Tuberculosis, in last decade, has garnered extreme attention because of acquired drug resistance by *Mycobacterium tuberculosis*. *M. tuberculosis* poses a major global health threat and requires advancements related to better diagnostic approaches. Thus, Bavda and Jain scrutinized Mycobacteriophage D29, for its potential to infect and eliminate several mycobacterial species. In order to test the viability of the phage after removal of crucial holin protein, Bacteriophage Recombineering of Electroporated DNA (BRED) approach was applied to create D29 holin knockout (D291gp11). Retained functional ability of the phage after removal of holin suggests the protein is dispensable for this phage, in particular. Moving further, Nair and Jain designed a novel diagnostic approach using D29 mycobacteriophage LysA endolysin's C-terminal domain (CTD) construct. CTD possesses the ability to selectively bind to *M. tuberculosis* and *Mycobacterium smegmatis* peptidoglycan (PG). Exploring this specific interaction, Green fluorescent protein (GFP) and glutathione-S-transferase (GST) were tagged to CTD forming CTD-GFP and CTD-GST constructs. These constructs were used to separate the mixtures containing Gram-positive and Gram-negative bacteria. These innovative studies added positively to the diagnostic and treatment cohort against *M. tuberculosis*.

The use of bacteriophages in the biological control of bacterial infections in plants has never been so common in comparison with their use in prevention and treatment of infections in humans and animals or in a food industry. This is also reflected in this Research Topic, in which two publications have targeted bacteriophages infecting plant pathogens and their thereof use. Thanh et al. morphologically, phenotypically and genetically characterized a EspM4VN bacteriophage to be used to control soft rot disease in crops infected with *Enterobacter* spp. Likewise, Lukianova et al. analyzed in detail the attachment of two bacteriophages, viz. PP99 and PP101 to the surface of the plant pathogen *Pectobacterium brasiliense*. The authors revealed that PP99 and PP101 phages use a similar mechanism by which they interact with bacterial surface polysaccharides. As well, the authors described in detail the molecular basis of this mechanism governing interaction of phage tail spike proteins with bacterial O-polysaccharide. This in turn is an important insight into phage adsorption mechanisms used by viruses to infect soft rot plant pathogens.

An interesting approach of using bacteriophages in food industry to prevent spoilage is presented by Feyereisen et al. who evaluated lytic bacteriophages against *Lactobacillus brevis* responsible for beer decay. The authors discovered and characterized in detail intact prophages in the genomes of *L. brevis* that can be potentially used as antimicrobial agents. The idea was to combine prophage induction followed by host killing with sanitizers and/or UV treatment to remove the *L. brevis* bacteria from beer production environment. This innovative approach may be used to reduce the chemical input in beer production and provide a proof-of-concept data to evaluate this idea also in other food-related production systems.

Another innovative approach to prevent death of farmed shrimps caused by *Vibrio* sp. Va-F3 infections under aquacultural conditions was described in the work of Chen et al.. The authors implemented an integrated workflow designed to develop a cocktail containing lytic bacteriophages, and evaluated it. Application of bacteriophage cocktail significantly reduced the death of the treated shrimps in comparison with the untreated control proving that such antibiotic-less therapies may be used to protect farmed animals in aquacultures.

It is believed that the blooming of the phage research will continue. The rediscovery of bacteriophages and their role in medicine, veterinary, food microbiology and agriculture has transformed the research area of minor importance to a broad and scientifically significant topic of the twenty-first century.

*Lastly, we would like to sincerely thank all the authors for their contribution to this thematic Research Topic, as well as acknowledge the excellent work of many reviewers for their critical assessments of the reviewed manuscripts*.

## Author Contributions

All authors listed have made a substantial, direct and intellectual contribution to the work, and approved it for publication.

## Conflict of Interest

The authors declare that the research was conducted in the absence of any commercial or financial relationships that could be construed as a potential conflict of interest.

## References

[B1] Abedon S.T. (2014). Phage therapy: eco-physiological pharmacology. Scientifica 2014:581639. 10.1155/2014/581639 25031881 PMC4054669

[B2] Hadley P. (1928). The Twort-D'Herelle phenomenon: a critical review and presentation of a new conception (homogamic theory) of bacteriophage action. J. Infect. Dis. 42, 263–434.

[B3] Van der Vlught R. A. A. Verbeek M. (2008). Bacteriophages: therapeuticals and alternative applications. Available online at: http://www.cogem.net/index.cfm/en/publications/publicatie/bacteriophages-therapeuticals-and-alternative-applications COGEM Report.

